# Perilipin5 protects against lipotoxicity and alleviates endoplasmic reticulum stress in pancreatic β-cells

**DOI:** 10.1186/s12986-019-0375-2

**Published:** 2019-07-30

**Authors:** Yunxia Zhu, Xiaoyan Zhang, Li Zhang, Mingliang Zhang, Ling Li, Deng Luo, Yuan Zhong

**Affiliations:** 1 Department of Geriatrics, Shanghai Jiao Tong University Affiliated Sixth People’s Hospital, No.600, Yishan Road, Shanghai, 200233 China; 2 Department of Endocrinology and Metabolism, Shanghai Jiao Tong University Affiliated Sixth People’s Hospital, Shanghai, 200233 China; 3 Department of Endocrinology, Tongji Hospital, Tongji University School of Medicine, Shanghai, 200065 China; 4 Department of Endocrinology, Renmin Hospital of Wuhan University, Wuhan, 430060 China

**Keywords:** Perilipin 5, Lipotoxicity, β-Cell function, Endoplasmic reticulum stress, Fatty acid oxidation

## Abstract

**Background:**

Chronic exposure of pancreatic β-cells to excess free fatty acids is thought to contribute to type 2 diabetes pathogenesis in obesity by impairing β-cell function and even leading to apoptosis. In β-cells, lipid droplet-associated protein perilipin 5 (PLIN5) has been shown to enhance insulin secretion by regulating intracellular lipid metabolism; the roles of PLIN5 in response to lipotoxicity remain poorly understood.

**Methods:**

INS-1 β-cells were transfected with PLIN5-overexpression adenovirus (Ad-PLIN5) and treated with palmitate. C57BL/6 J male mice were fed with high fat diet and tail intravenous injected with adeno-associated virus overexpressing PLIN5 (AAV-PLIN5) in β-cells.

**Results:**

Our data showed that palmitate and PPAR agonists including WY14643 (PPARα), GW501516 (PPARβ/δ), rosiglitazone (PPARγ) in vitro all induced PLIN5 expression in INS-1 cells. Under palmitate overload, although upregulating PLIN5 promoted lipid droplet storage, it alleviated lipotoxicity in INS-1 β-cells with improved cell viability, cell apoptosis and β-cell function. The protection role of PLIN5 in β-cell function observed in cell experiments were further verified in in vivo study indicated by mitigated glucose intolerance in high fat diet fed mice with β-cell-specific overexpression of PLIN5. Mechanistic experiments revealed that enhanced FAO induced by elevation of PLIN5, followed by decreased ER stress may be a major mechanism responsible for alleviation of lipotoxicity observed in the present study.

**Conclusions:**

Our finding substantiated the important role of PLIN5 in protection against lipotoxicity in β-cells.

**Electronic supplementary material:**

The online version of this article (10.1186/s12986-019-0375-2) contains supplementary material, which is available to authorized users.

## Background

The prevalence of type 2 diabetes (T2DM), a chronic metabolic disorder, has been increasing steadily, partially due to the rising obesity rates. Excess adiposity and dyslipidemia commonly seen in obesity not only induce insulin resistance but also directly damage β-cell function, a fundamental defect in diabetes [1]. Indeed, chronic exposure of pancreatic β-cell to free fatty acids (FFA), accompanied with obesity, may impair insulin secretion and ultimately lead to β-cell apoptosis in vivo and ex vivo, a phenomenon termed “lipotoxicity” [2, 3]. Although the molecular mechanism underlying lipotoxicity remain incompletely understood, endoplasmic reticulum (ER) stress has been considered to be an important factor mediating lipotoxicity in islet β-cells [4, 5]. ER stress in β-cells is known as an imbalance between the protein folding capacity and the increased demands of insulin production and secretion. To restore ER homeostasis, cells trigger the unfolded protein response (UPR) and failure of this process leads unresolved ER stress. There are three major signaling arms of the UPR initiated by the serine/threonine-protein kinase/endoribonuclease IRE1, the transcription factor activating transcription factor 6 (ATF6), and protein kinase RNA-like endoplasmic reticulum kinase (PERK). ER protein folding capacity is increased by XBP-1-medicated upregulation of ER chaperones including immunoglobulin heavy chain-binding protein (BiP) [6]. When ER stress is prolonged or excessive, apoptotic cell death is executed by C/EBP homologous protein (CHOP) and other components of the apoptotic machinery [7]. There is accumulating evidence that some of these ER stress markers including XBP-1, BiP, and CHOP were elevated not only in chronic palmitate (PA) treated β-cell lines but also in pancreatic islets of patients with T2DM [8–11].

Excessive FFA are mainly stored in the form of triglyceride (TG) in lipid droplet (LD) in most of mammalian cells. LD consist of a score of neutral lipids (i.e., triglycerides and/or sterol esters) surrounded by a phospholipid monolayer in which are embedded proteins named LD-associated proteins [12]. In mammalian cells, LD act as a fuel supplier during periods of energy needs but also a lipolytic barrier to avoid cellular lipotoxicity by regulating LD lipolysis and therefore plays a critical role in maintaining lipid metabolism homeostasis and this metabolic regulation of LD was mediated by LD-associated proteins [13, 14].

The perilipin family proteins (PLINs) were the major LD-associated proteins that encompass five members, from PLIN1 to PLIN5. In contrast to PLIN1, which is mostly highly expressed in white adipose tissue, PLIN5 (also named LSDP5, OXPAT, or MLDP) is mainly expressed in tissues with high oxidative capacity such as red muscle, heart, liver and brown adipose [15]. As a LD-associated protein, previous studies including ours have suggested its importance in the regulation of lipid metabolism [16–18]. In the unstimulated or rested conditions, by interacting with lipases to suppress triglyceride breakdown and promotes LD formation, PLIN5 can limit the production of bioactive lipid metabolites and cellular stress signals and thereby prevents tissue dysfunction [17, 18]. Hence, PLIN5 acts as a protective factor against lipotoxicity and modulates ER stress in liver and skeletal muscle [14, 17, 19, 20]. While, under the stimulated conditions such as muscle contraction or PKA treatment, PLIN5 will sharply boost lipolysis and facilitate fatty acid oxidation (FAO) to meet metabolic demand in skeletal muscle [14]. However, how does PLIN5 act in β-cells, the central of diabetes incidence, remain largely unknown. Up to date, there is only one associated paper in which PLIN5 is reported to support postprandial insulin secretion by facilitating FA mobilization including lipolysis and FAO upon cAMP stimulation as seen postprandially [21]. Thus, based on the current understanding of PLIN5 in other metabolic tissues, we hypothesize that PLIN5 may also play a critical role in protection β-cell from lipotoxicity and influence ER stress. In the present study, the functional role of PLIN5 was studied in vitro in INS-1 β-cells and in vivo in mouse pancreatic β-cells. Our data here verified our assumption that upregulation of PLIN5 alleviated PA-induced ER stress partially by enhancing FAO and may hence decreased β-cell toxicity from lipid overload.

## Methods

### Animal studies

Male C57BL/6 J mice (8–10 weeks old) were maintained on a 12-h light/dark cycle with free access to food and water. After 1 week of acclimation, mice were fed chow diet (CHOW; Shanghai SLAC Laboratory Animal Co. Ltd., Shanghai, China) or a high fat diet (HFD; Research Diets Inc., New Brunswick, NJ, USA) containing 10% or 60% kcal fat for 5 months. For adeno-associated virus (AAV) transfection in mice, AAV2/8 vector carrying cDNA for human PLIN5 (AAV-PLIN5) under mouse insulin-II promoter were custom made at Shanghai AsiaVector Biotechnologies. cDNA fragment of PLIN5 was PCR amplified using the following primers: forward 5′-CCGGAATTCATGGACCAGAGAGGTGAAGACACCA-3′, reverse 5′-CCGCTCGAGGAAGTCCAGCTCTGGCATCATTGTG-3′ and then ligated into AAV2/8-MIP vector containing the mouse insulin 2 promoter and GFP. The details of AAV are available in Additional file 1: Fig. S1. AAV-PLIN5 were produced by co-transfection with the helper/packaging plasmid in HEK-293 T cells. Virus was purified by double CsCl gradient centrifugation and viral genomes were quantified by dot blot analysis. The AAV-MIP was used as the control. The concentration of viral vector stock was 2.5 × 10^12^ viral genome/ml. After 5-month dietary manipulation, AAV-PLIN5 or AAV-MIP was delivered to HFD mice by tail vein injection at 5 × 10^11^ viral genome per mouse. CHOW mice were injected with saline as the control group. 1 week after injection, fasting (12 h) and fed glucose (2 h) levels were determined from tail vein blood with a glucometer (FreeStyle, Alameda, CA, USA). 10–14 days after injection, glucose tolerance tests (GTT) and insulin tolerance tests (ITT) were conducted. For GTT, mice were fasted for overnight (16 h) and then injected i.p. with D-glucose at 1.5 g/kg of body weight. For ITT, mice were performed after 6 h fasting and injected i.p. with human regular insulin (Novo Nordisk, Denmark) at 0.75 IU/kg of body weight. Blood glucose was measured from tail vein blood at the indicated time interval after the injection using a glucometer (FreeStyle). The areas under the curves for blood glucose in GTT (AUC_g_) and ITT (AUC_itt_) were calculated. Serum insulin at 0, 15, 30 min after GTT (Mercodia AB, Sweden) and fasting serum FFA (R&D Systems, Minneapolis, MN, USA) were measured according to manufacturers’ instructions. At the end of the study, mice pancreatic islets were obtained as described previously [22]. To further investigate the expression pattern of PLIN5 in islets under different pathophysiological condition, the obesity mouse models ob/ob mice (16-week-old, purchased from The Jackson Laboratory) were used. All experimental procedures and protocols were approved by the Animal Care and Use Committees of Shanghai Jiaotong University Affiliated Sixth People’s Hospital, Shanghai, China.

### Cell treatments and adenovirus transfection

The rat insulinoma INS-1 cell line was kindly provided by Dr. Christopher B. Newgard (Duke University Medical Center) and maintained in RPMI 1640 as described [23]. INS-1 cells between passages 20–30 were used, which possessed robust glucose-stimulated insulin secretion properties. Recombinant adenovirus encoding full-length rat PLIN5 (Ad-PLIN5) were custom made at Shanghai AsiaVector Biotechnologies (China). cDNA fragment of PLIN5 was chemical synthesized and then ligated into the pTRACK-CMV vector. The vector was linearized by Pme I digestion and transfected into 293A cells for virus production and amplification. Adenovirus overexpressing GFP (Ad-GFP) was the control. INS-1 cells were infected with adenovirus at a multiplicity of infection (MOI) of 20–40 for 24 h, and adenovirus infections under these conditions did not show significant effects on cellular viability as analyzed using the Cell Counting Kit-8 (CCK-8; Dojindo, Kumamoto, Japan). For fatty acid treatment, we incubated INS-1 cells for 24 h in RPMI 1640 medium with either PA (Sigma-Aldrich, St Louis, MO, USA) precoupled to BSA or BSA alone. Briefly, PA was dissolved at 100 mM in ethanol to make the stock solution and the stock solution was then diluted in the culture medium to which fatty acid-free BSA had been added, in a 1:3 M ratio, to prepare BSA-conjugated PA. The BSA-conjugated PA was added to cells at a final concentration of 0.5 mM. For PPAR agonist treatment, the cells were washed twice with phosphate-buffered saline (PBS) and incubated in fresh RPMI-1640 medium containing 11.1 mM glucose. The replacement media supplemented with either 10 μM WY-14643 (agonist of PPARα), or 10 μM GW501516 (agonist of PPARβ/δ), or 10 μM rosiglitazone (agonist of PPARγ), which all bought from Alexis Biochemicals, Lausen, Switzerland. The cells were incubated for 24 h and then PLIN5 protein level was detected by immunoblotting. For lipid oxidation inhibition, etomoxir (ET, Sigma), the inhibitor of carnitine palmitoyltransferase 1 (CPT-1) and the rate-limiting enzymes in fatty acid β-oxidation was used in the concentration of 50 μM.

### Plasmid and siRNA transfection

CPT-1A overexpression plasmid and the control plasmid were made at GeneChem (Shanghai, China). Briefly, rat cDNA encoding CPT-1A was chemical synthesized and sub-cloned into GV146 vector between XhoI and EcoRI restriction sites and the construct was verified by sequencing. The siRNA sequences used for silencing rat PLIN5 (si-PLIN5) and the scrambled siRNA (si-Con) were also supplied by GeneChem. The sequences of si-PLIN5 were 5′- GGGACUAGACAAAUUGGAATT − 3′ (sense) and 5′-UUCCAAUUUGUCUAGUCCCTT-3′ (antisense). And the scrambled siRNA were 5′- GUACUUUUGUGUAGUACAATT − 3′ (sense) and 5′-UUGUACUACACAAAAGUACTT-3′ (antisense). Different plasmids and siRNA were co-transfected into INS-1 cells according to indicated groups using Lipofectamine 2000 (Invitrogen), and 48 h later cells were treated.

### Dual luciferase reporter gene assay

The PLIN5 luciferase reporter vector was constructed by Genechem (Shanghai, China). Briefly, rat cDNA encoding PLIN5 promoter was amplified using the following primers: forward 5′- TTTCTCTATCGATAGGTACCTGACAACCAGCTAACCGCAGTGGC-3′, reverse 5′- CTTAGATCGCAGATCTCGAGCTGAGTGCAGAGCTCAGACTCTGC-3′. Then, the amplified fragment was sub-cloned into GV238 vector (Genechem). After co-transfecting with PLIN5-luciferase reporter vector (PLIN5-Luc, 750 ng) and the control reporter plasmid pRL-TK (50 ng) for 6 h, INS-1 cells were treated with three PPAR agonists for 24 h, respectively. Then, cells were harvested and lysed and luciferase reported activity was measured using the Dual-Luciferase Assay Kit (Promega, USA). The activity of firefly luciferase was normalized to that of Renilla luciferase.

### Lipid staining

For the LD analysis, cell lipids were stained with Nile red (Sigma, MO, USA) and were observed by confocal microscopy. Briefly, INS-1 cells were first washed three times with PBS and then fixed in 10% formaldehyde in PBS for 20 min. After three washes, fixed cells were stained with the Nile red solution (1 μg/mL) for 10 min at room temperature, followed by three washes with water. Cells were then observed using a confocal microscope with a 200× objective and images were analyzed using Image-Pro Plus 5.0 software (Media Cybernetics Inc., MD, USA) for semiquantitative determination of neutral lipids in Nile red-stained cells. The calculated values were showed as the LD-positive area (μm^2^) per cell and mean values were calculated for fluorescence on three coverslips of cell from three separate experiments and averaged to provide values for each treatment group.

### Cell viability assay

The cytotoxicity of PA was measured in triplicate by using CCK-8 assay. CCK-8 contains a highly water-soluble tetrazolium salt WST-8 [2-(2-methoxy-4-nitrophenyl-3-(4-nitrophenyl)-5-(2,4-disulfophenyl)-2H-tetrazolium] reacting with mitochondrial dehydrogenase to produce an orange water soluble formazan dye. Prior to the analysis, the INS-1 cells were plated in 24-well cell culture plates at a density of 5 × 10^4^ cells per well. After the treatments such as adenovirus infection, PA or PPAR agonist incubation, each well was added to 50 μl of CCK-8 solution and the plates were incubated at 37 °C for 4 h to allow formation of formazan crystals. Absorbance at 450 nm (OD 450) was then read by a microplate reader (Tecan Infinite 200, Mannedort, Switzerland). Cells incubated with culture medium alone were used to determine 100% viability and were included as a nontreatment control to allow estimation of the percent viability of treated cell samples.

### Cells apoptosis by flow cytometry

Cell apoptosis was measured by flow cytometric analysis. Briefly, after treatments, the cells were trypsinized, washed with PBS, and suspended in 195 μl binding buffer. Then, cells were incubated with 5 μl Annexin V-FITC for 15 min and 5 μl propidium iodide (Beyotime, China) for 5 min at 4 °C in the dark. The cells were then analyzed on a FAC Scan flow cytometer (Becton-Dickinson, CA, USA) to quantify apoptosis.

### Glucose-stimulated insulin secretion (GSIS)

INS-1 cells were treated with adenovirus for 24 h in RPMI 1640 medium supplemented with 10% fetal bovine serum (FBS), after which 0.5 mM PA was added to the medium for another 12 h or 48 h. Cells were starved for 2 h in glucose-free RPMI 1640 and then were washed twice with glucose-free Krebs-Ringer bicarbonate (KRB) buffer (pH 7.4). GSIS was investigated as described previously, with minor modifications [20]. Briefly, the cells were further stimulated with either low glucose (2.5 mM) or high glucose (25 mM) for 1 h at 37 °C. The supernatants were collected for insulin determination, and cellular insulin contents were determined from acid-ethanol extracts. Insulin concentration was measured using a rat insulin ELISA kit (Mercodia Co., AB, Uppsala, Sweden) according to the manufacturer’s instructions after appropriate dilution. Total protein was extracted with RIPA lysis buffer supplemented with 1 mM phenylmethyl sulfonylfluoride, and the protein concentration was determined using a BCA protein assay kit (Pierce Biotechnology, Inc., Rockford, IL, USA). The levels of insulin secretion were normalized against the respective protein content. Insulin secretion following stimulation with 2.5 mM and 25 mM glucose was defined as basal insulin secretion and GSIS, respectively.

### Antibodies and immunoblotting

Proteins were extracted and immunoblotted as described previously [22]. The following primary antibodies included FAS, ATGL, glucagon-like peptide-1 receptor (GLP-1R), phospho-ERK1/2 (p-ERK1/2), total ERK1/2, P70^S6K^, Bcl-2, phospho-mTOR (p-mTOR), total mTOR which were all purchased from Cell Signaling and PLIN5 from Progen Biotechnik, ACO from Abcam, CHOP from Immunoway, CPT-1 and BiP from Santa Cruz. All the secondary antibodies and internal reference GAPDH were all purchased from Santa Cruz. Immunoreactive bands were visualized with an ECL reagent kit (Millipore). Optical densities of each band were calculated and analyzed by using Image J analysis software.

### Quantitative real-time RT-PCR (Q-PCR) analysis

Total RNA from INS-1 cell line was isolated using TRIzol reagent (Invitrogen, Carlsbad, CA, USA). First-strand cDNA was synthesized with Moloney murine leukemia virus (M-MLV) reverse transcriptase and random hexamer primers (Invitrogen, Carlsbad, CA, USA). Gene expressions were analyzed using the SYBR Green PCR system, following the manufacturer’s recommendations (Applied Biosystems, Foster City, CA, USA). *Cyclin D1*, *Cyclin D2*, *spliced XBP-1* (*XBP-1(s)*), *sterol-regulatory element binding protein-1c* (*SREBP-1c*), *CHOP, BiP, and PLIN5* were detected using primers indicated in Table 1. 18S rRNA was used as an internal control for all genes.

**Table 1:** Primers of quantitative RT-PCR analysis

Name	Forward/reverse primer sequences
PLIN5	AGGGCTACTTTGTGCGTCTG; TTTGGGTGATGGAAAGTAGG
SREBP-1c	GCTGAGTGCCCTGAACCTG; CCCATCCACGAAGAAACG
XBP-1(s)	GAGTCCGCAGCAGGTG; GCGTCAGAATCCATGGGA
Cyclin D1	TCTACACTGACAACTCTATCCG; TAGCAGGAGAGGAAGTTGTTGG
Cyclin D2	AGACCTTCATCGCTCTGTGT; TAGCAGATGACGAACACGCC
BiP	GCGGATCAAGGTGAAGAAAGG; CGCAAAGTCTACCCACAGGAAC
CHOP	AAGGAGAAGGAGCAGGAGAATG; ATGCGGTCGATCAGAGCC
18S rRNA	AGGGGAGAGCGGGTAAGAGA; GGACAGGACTAGGCGGAACA

### Statistical analyses

All quantitative data were presented as means ± SEM and cell experiments were performed at least three times. Differences of numeric parameters between two groups were assessed using Student’s *t*-tests. To compare data sets of more than two groups, we used one-way ANOVA followed by Bonferroni’s multiple comparison tests. Two-way ANOVA determined significance for GTT and ITT. *P* < 0.05 (two-tailed) was considered significant.

## Results

### Modulation of PLIN5 expression in INS-1 pancreatic β-cells

**Fig. 1:**
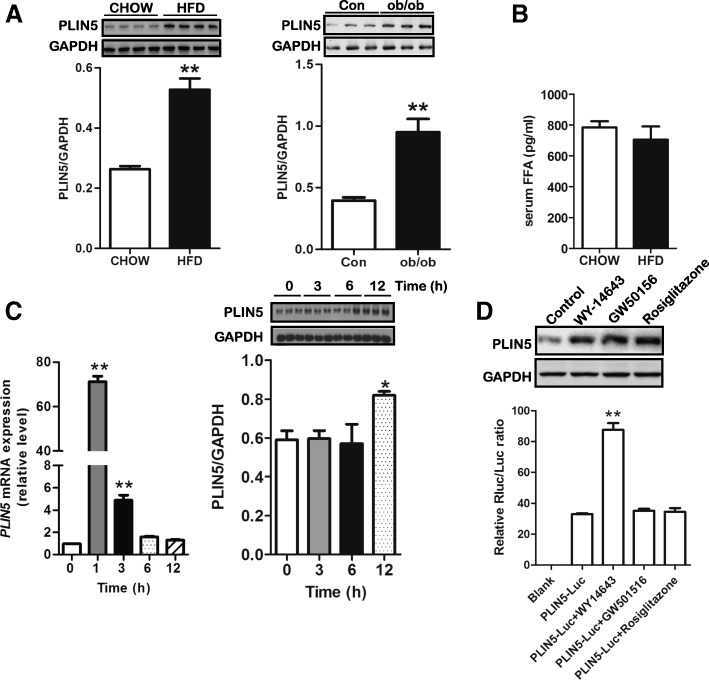
Regulations of PLIN5 expression in pancreatic islets and INS-1 β-cells. **a** Upregulation of islet PLIN5 protein expression in high fat diet (HFD) fed mice (left, *n* = 4 pooled samples for each group) and ob/ob obesity mice (right, *n* = 3 samples for each group) compared with chow diet fed (CHOW) mice or control mice (Con). Pancreatic islet PLIN5 protein expression was detected from C57BL/6 J mice treated with chow food or HFD for 5 months and 16-week old ob/ob mice or the control mice. **P < 0.05 vs CHOW or control (Con). **b** Serum FFA levels were comparable between CHOW mice and HFD mice (*n* = 8 for each group). After 5-month food manipulation, serum FFA concentrations were measured using a commercial kit. **c** Time course of palmitate (PA) treatment on gene (left) and protein (right) expression of PLIN5. INS-1 cells were treated with 0.5 mM PA for 0, 1, 3, 6, and 12 h (*n* = 3 for each group in three independent experiments). Abundance of PLIN5 was detected by quantitative real-time RT-PCR (Q-PCR) and Western blot. For Q-PCR, the mRNA level was expressed as the percentage of control (0 h). Values are means ± SE. **P < 0.01 vs. *0 h.* **d** PLIN5 is a PPAR target gene in INS-1 β-cells (*n* = 3 for each group in three independent experiments). Cells were exposed to 10 μM WY-14643 (PPARα agonist) or 10 μM GW501516 (PPARβ/δ agonist) or 10 μM rosiglitazone (PPARγ agonist) for 24 h and expression of PLIN5 was monitored by Western blot (upper). A luciferase reporter assay was conducted to verify the interactions between PLIN5 and PPARs (down). INS-1 cells were transfected with PLIN5-luciferase reporter vector and then treated with three PPAR agonists for 24 h, respectively. After that, luciferase activity was measured which is the ratio between firefly luciferase and Renilla control luciferase. Values are means ± SE. **P < 0.01 vs. *PLIN5-Luc*

Recently, PLIN5 was confirmed to be an LD protein in both human and murine islets and altering expression level of PLIN5 can impact insulin secretion by regulating lipolysis [21]. However, little is known about its regulation. Here, we analyzed the effect of nutrition overload and PPAR agonists treatment on its level. After 5-month of HFD, C57BL/6 J mice became obese (data not shown). In islets, there was a significant upregulation in PLIN5 protein level among HFD mice and ob/ob mice although the serum FFA level was comparable in CHOW group and HFD group (Fig. 1a, b). As we known, PLIN5 expression was elevated in myocytes and hepatocytes, when exposed to FFA [24, 25], we next examined PLIN5 expression in pancreatic β-cells exposed to PA. INS-1 pancreatic β-cells were treated with 0.5 mM PA for different periods of time (0, 1, 3, 6, and 12 h). As shown in Fig. 1c, incubation with PA significantly increased PLIN5 expression in both mRNA and protein levels. The Q-PCR data showed that PLIN5 mRNA levels increased sharply after 1 h treatment and led to about 70-fold increase in INS-1 cells. At longer times, the level of PLIN5 mRNA dramatically declined almost basal level by 12 h. The Western blot data confirmed the upregulation of PLIN5 by PA incubation for 12 h. PLIN5 has been described as a PPAR-target gene in non-insulin producing cells such as myocytes, adipocytes and hepatocytes [15, 26, 27]. Here, we elaborate on the regulation of PLIN5 by PPARs in INS-1 β-cells. We cultured INS-1 cells in medium containing one of the following agonists respectively: WY14643 (PPARα), GW501516 (PPARβ/δ), rosiglitazone (PPARγ). The GAPDH protein was used as a loading control in the experiment. As showed in Fig. 1d, PLIN5 protein was upregulated by WY14643, GW501516, and rosiglitazone treatment. Considering that FFAs are also natural ligands that may increase PPARs transcriptional activity, we used luciferase reporter assays to detect the direct interaction between PLIN5 and PPARs and to investigate whether the induction of PLIN5 by PA is dependent on PPARs activation. As shown in Fig. 1d, WY14643 but not GW501516 or rosiglitazone treatment in INS-1 β-cells led to a significant increase of luciferase gene reporter activity showing that PPARα are direct regulators of *PLIN5*. Hence, the upregulation of PLIN5 by PA may dependent on PPARα activation in INS-1 cells. And the induction role of PLIN5 expression by PPARβ/δ and/or PPARγ activation is indirect which needs to be investigated in the future study.

### Overexpression of PLIN5 increases lipid storage in INS-1 β-cells

**Fig. 2:**
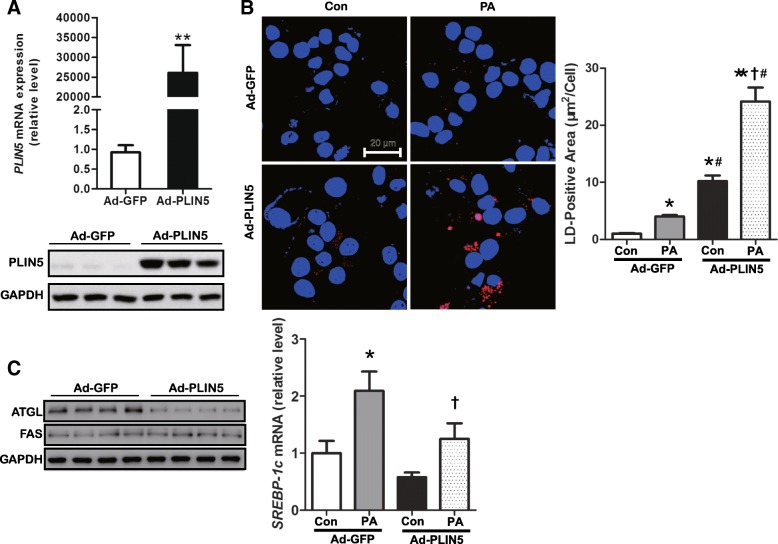
PLIN5 and PA synergically accelerate LD formation. **a** Ad-PLIN5 transfection induced PLIN5 overexpression in INS-1 cells at mRNA (upper) and protein (down) level. **b** INS-1 cells were transfected with Ad-GFP or Ad-PLIN5, and 0.5 mM palmitate (PA) was loaded for 12 h. Subsequently, cells were fixed, stained with Nile red (red) to detect LD, and observed by confocal microscopy with the magnification of 200×. Nucleus stained with DAPI (blue). Nile red area was evaluated semiquantitatively by Image-Pro Plus 5.0 software from random 20 high-power fields and standardized by cell numbers. **c** The levels of ATGL, FAS were examined by western blot analysis and *SREBP-1c* by Q-PCR. Values are means ± SE from three independent experiments, each conducted in triplicate. * *P* < 0.05, **P < 0.01 vs. Ad-GFP group. # *P* < 0.05, vs. Ad-GFP + PA group. †*P* < 0.05, vs. Ad-PLIN5 group

PLIN5 has been shown to closely correlate with lipid storage in some kinds of cells such as hepatocytes, myocytes, and cardiomyocytes [18, 28, 29]*.* In fasted islets, elevated PLIN5 expression and high TG content was observed recently [21]. To better understand the functional role of PLIN5 in lipid storage of β-cells, we performed PLIN5 overexpression in INS-1 cells by transfection with PLIN5 adenovirus (Ad-PLIN5) and cells transfected with GFP adenovirus (Ad-GFP) as the control group. PLIN5 upregulation was confirmed by Q-PCR and Western blot (Fig. 2a). As expected, PLIN5 overexpression increased LD-positive area detected by Nile red staining after 12 h PA treatment (Fig. 2b). To further investigate the mechanism of PLIN5 induction in lipid metabolism preliminarily, we detected the expression of molecules involved in both lipolysis and lipogenesis including ATGL, SREBP-1c and FAS. FAS and SREBP-1c are both major factors involved in lipid synthesis, while ATGL play a key role in LD degradation [30]. We found that ATGL but not FAS protein level was decreased by upregulating PLIN5 (Fig. 2c). Q-PCR data showed that PLIN5 had no effect on the expression of *SREBP-1c* (Fig. 2c). Thus, our data indicated that PLIN5 overexpression may exaggerate LD formation by decreasing lipid degradation which is associated with ATGL.

### Overexpression of PLIN5 improves GSIS of INS-1 cells impaired by prolonged PA treatment

**Fig. 3:**
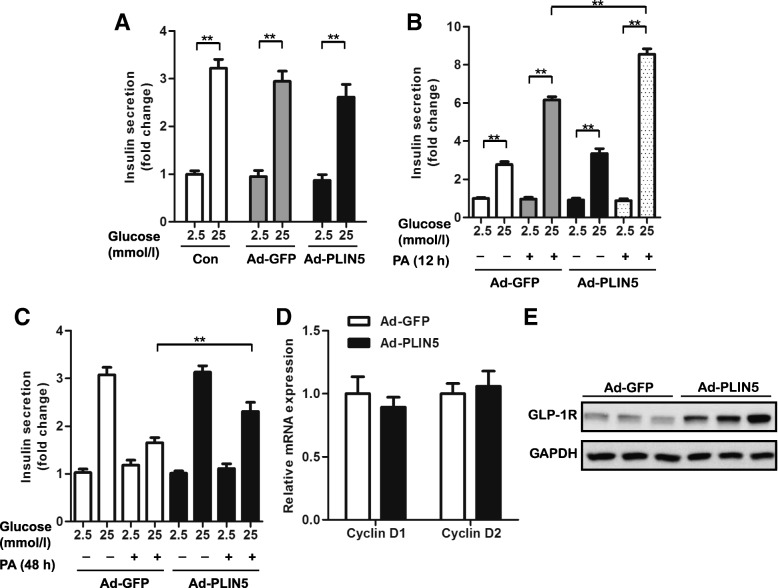
Overexpression of PLIN5 improved GSIS in long-term PA treated INS-1 cells. **a** GSIS of untransduced INS-1 cells and those transduced with Ad-GFP or Ad-PLIN5. **b** GSIS with or without 0.5 mM PA for 12 h in Ad-PLIN5 INS-1 cells compared with Ad-GFP control. **c** GSIS with or without 0.5 mM PA for 48 h in Ad-PLIN5 INS-1 cells compared with Ad-GFP control. Data expressed as the fold change taking GSIS of untransduced INS-1 cells (A) or Ad-GFP at 2.5 mM glucose (B and C) as 100%. **d** Q-PCR analysis of the mRNA levels of *Cyclin D1* and *Cyclin D2* in the INS-1 cells transduced with Ad-GFP or Ad-PLIN5. **e** Immunoblotting analysis of the levels of GLP-1 receptor (GLP-1R) in the INS-1 cells transduced with Ad-GFP or Ad-PLIN5. Values are means ± SE from three independent experiments, each conducted in triplicate. **P < 0.01, vs. indicated group

It is known that lipotoxicity can ultimately lead to β-cell dysfunction and PLIN5 has been reported to play a protective role against lipotoxicity in skeletal muscle and liver [14, 17]. We then sought to determine the role of PLIN5 on β-cell function in the context of PA treatment. Adenovirus-mediated expression of GFP and PLIN5 did not interfere with GSIS in INS-1 cells (Fig. 3a), so did cellular insulin content (data not shown). As shown in Fig. 3b, upregulation of PLIN5 augmented GSIS induced by 12 h PA administration. While, as PA overload prolonging (48 h), GSIS was significantly impaired in INS-1 cells. Obviously, PLIN5 overexpression effectively rescued the GSIS, indicating that PLIN5 plays an important role in the protection β-cell from PA induced insulin secretion dysfunction (Fig. 3c). PLIN5 was not involved in the regulation of β-cell proliferation as the gene level of *Cyclin D1* and *Cyclin D2* were comparable between Ad-GFP and Ad-PLIN5 (Fig. 3d). Surprisingly, the expression of GLP-1R, the receptor of an endogenous incretin hormone which can promote insulin secretion in the pancreas when glucose levels are elevated, was significant induced by overexpressing PLIN5 in INS-1 β-cells (Fig. 3e).

### Overexpression of PLIN5 improves glucose intolerance of obese mice induced by HFD feeding

**Fig. 4:**
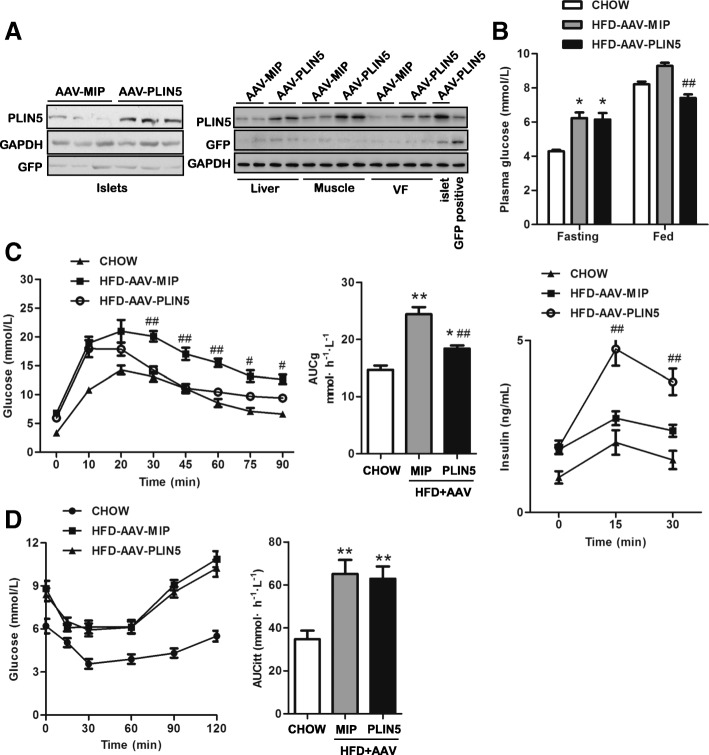
Upregulation of PLIN5 in β-cells improves glucose tolerance without interfering with insulin sensitivity in HFD mice. **a** PLIN5 overexpression was confirmed in β-cells of mice (*n* = 5 for each group). Western blots showed that most of the AAV were delivered to β-cells (left) with a few also entering liver, muscle, and visceral fat (right) in mice. **b** Fasting and fed glucose levels of mice (*n* = 8 for each group). After HFD treatment for 5 months, AAV-PLIN5 and AAV-MIP were delivered to HFD mice and Chow mice was injected with saline by tail vein. One week after injection, fasting (12 h) and fed glucose (2 h) levels were determined from tail vein blood. **c** GTT of CHOW, HFD-AAV-MIP, and HFD-AAV-PLIN5 mice (*n* = 5–8 for each group). Ten to fourteen days after tail vein injection, GTT was conducted. Serum insulin at 0, 15, and 30 min after GTT were measured in different groups. **d** ITT of CHOW, HFD-AAV-MIP, and HFD-AAV-PLIN5 mice. AUC of ITT was calculated (*n* = 8 for each group). Values are means ± SE. AUCg: area under the curve of the GTT. GTT: glucose tolerance test. ITT: insulin tolerance test. *P < 0.05, **P < 0.01, vs. CHOW; # *P* < 0.05, ## *P* < 0.01, vs. HFD-AAV-MIP

In order to verify the protection role against lipotoxicity in β-cell function in vivo, β-cell-specific PLIN5 overexpression mouse model was established by tail vein injection with AAV-PLIN5 (Fig. 4a). First, C57BL/6 J male mice were induced obesity by HFD feeding for 5 months. Then, obese mice were further delivered either AAV-PLIN5 or AAV-MIP, and CHOW mice injected with saline was used as the control group. 1 week after injection, plasma glucose was measured in fasted and fed mice (2 h) (Fig. 4b). The two HFD groups both showed increased fasting glucose compared with CHOW group, while it was comparable between the two HFD groups. For fed glucose, HFD-AAV-PLIN5 group was apparently lower than that of HFD-AAV-MIP group but not that of CHOW mice. It seemed that β-cell-specific PLIN5 overexpression contributed to the improvement of glucose tolerance in vivo. To confirm this, we subjected mice to a GTT and in vivo GSIS assay. AAV transfection had no effect on GTT (data not shown). In line with our in vitro data, PLIN5 overexpression induced a significant improvement of glucose tolerance in HFD mice (Fig. 4c). Accordingly, increased insulin secretion in response to the glucose load was observed in HFD-AAV-PLIN5 mice, as showed in Fig. 4c. It has been described that a global or muscle specific PLIN5 deficiency alleviated muscle insulin resistance in mice [14, 19]. Therefore, we next asked whether altering PLIN5 expression in islets would influence systemic insulin resistance detected by ITT as a few AAV were also detected in insulin targeted tissues such as liver, muscle and visceral fat. As shown in Fig. 4d, insulin action was comparable between the two HFD groups. Taken together, these data demonstrate that upregulating PLIN5 in β-cell was able to improve the β-cell dysfunction induced by HFD, while exerted no effects on insulin action.

### Overexpression of PLIN5 improves INS-1 cell viability and decreases apoptosis under lipotoxic stress

**Fig. 5:**
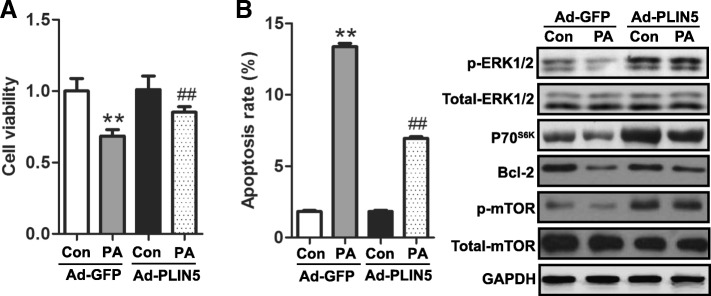
Upregulation of PLIN5 alleviated PA-induced cytotoxicity and apoptosis in INS-1 cells. INS-1 cells transfected with Ad-GFP or Ad-PLIN5 were incubated with control medium or 0.5 mM PA for 24 h. **a** Protective effect of PLIN5 on PA-induced impairment in cell viability. Cell viability was determined using CCK-8 assay. **b** Overexpression of PLIN5 protected against PA-induced apoptosis. Cell apoptosis was measured by flow cytometry (left). Apoptosis associated molecules expressions were determined by Western blots (right). Data were expressed as the fold change taking Ad-GFP as 100%. Values are means ± SE from three independent experiments, each conducted in triplicate. **P < 0.01, vs. Ad-GFP; ##*P* < 0.01 vs. Ad-GFP + PA

Next, we studied the possible role of PLIN5 in regulating cell viability detected by CCK-8 assay. As expected, PLIN5 overexpression significantly rescued impaired INS-1 cell viability induced by PA treatment for 24 h, while exhibited no effect on cell viability under regular culture condition (Fig. 5a). Flow cytometry analysis revealed that cell apoptosis because of PA was also partially rescued (Fig. 5b). To further assess the signaling molecules associated with PA-mediated β-cell apoptosis, protein levels of ERK1/2, P70^S6K^, Bcl-2 and mTOR were analyzed by Western blot. As shown in Fig. 5b, PA induced significant downregulation in p-ERK1/2, P70^S6K^, Bcl-2 and p-mTOR levels. However, overexpression of PLIN5 increased these proteins content under the condition of PA treatment.

### Overexpression of PLIN5 alleviates lipid overload induced ER stress partially by enhancing FAO

**Fig. 6:**
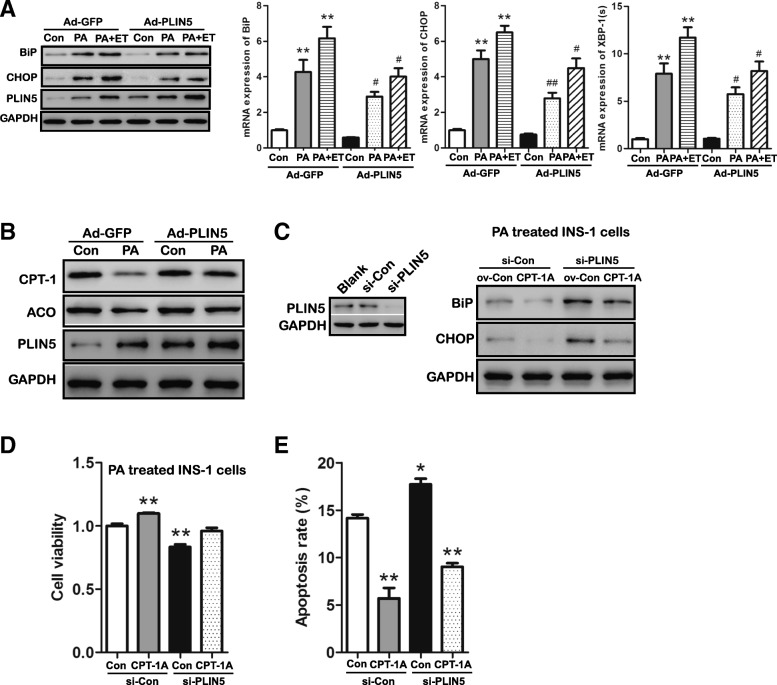
Upregulation of PLIN5 attenuated PA-induced ER stress partially by enhancing FAO in INS-1 cells. **a** Expression of BiP and CHOP was examined by Western blot (left) and Q-PCR (right). INS-1 β-cells transduced by Ad-GFP or Ad-PLIN5 were treated with 0.5 mM PA alone or preincubation with etomoxir (ET) for 30 min and then incubated with 0.5 mM PA for 24 h. *P < 0.05, **P < 0.01, vs. Ad-GFP; #*P* < 0.05, ##*P* < 0.01 vs. Ad-GFP + PA. **b** Expression of CPT-1 and ACO was examined by Western blot. INS-1 β-cells were transduced by Ad-GFP or Ad-PLIN5 and then treated with control medium or 0.5 mM PA for 24 h. **c** Expression of BiP and CHOP was examined by Western blot (right). PLIN5 knockout induced by si-PLIN5 transfection was confirmed by Western blot (left). INS-1 β-cells co-transduced by different plasmids (CPT-1A overexpression plasmid and the control vector) and/or siRNA (PLIN5 siRNA and the control siRNA) were treated with 0.5 mM PA for 24 h. **d** Protective effect of CPT-1A overexpression on cell viability in PA treated INS-1 cells with PLIN5 deletion. Cell viability was determined using CCK-8 assay. **P < 0.01, vs. si-Con. **e** Protective effect of CPT-1A overexpression on PLIN5-induced improvement in cell viability. Cell viability was determined using CCK-8 assay. *P < 0.05, **P < 0.01, vs. si-Con. Values are means ± SE from three independent experiments, each conducted in triplicate

In lipotoxic conditions, ER stress signaling is triggered in INS-1 β-cells [10, 31]. In the present study, we assayed for the key molecules that involve in the UPR signaling pathways. As shown in Fig. 6a, prolonged PA incubation (24 h) induced ER stress in INS-1 β-cells indicated by increased protein expression of CHOP, BiP and gene expression of *XBP-1(s).* However, upregulating PLIN5 compromised this induction effect of PA which suggesting the mitigation of ER stress induced by PA overload (Fig. 6a).

PLIN5 is a regulator of FAO [15, 28, 32], and enhanced FAO has been found to attenuate PA-induced ER stress in β-cells [33, 34]. Here, we aim to investigate whether PLIN5 alleviates PA-induced ER stress by enhancing FAO. As shown in Fig. 6b, the significant downregulation of two key enzymes involved in lipid oxidation, CPT-1 and ACO, was found in response to PA solo treatment which suggesting decreased FAO in β-cells. Strikingly, PLIN5 gain-of-function notably restored the expression of CPT-1 and ACO which suggesting the increased FAO level (Fig. 6b). To further verify the role of PLIN5 in attenuating lipid oxidation-associated ER stress, we used ET (an inhibitor of CPT-1) and CPT-1A (the main form of CPT-1 in pancreatic β-cells) overexpression plasmid to block and induce FAO, respectively. As shown in Fig. 6a, preincubation with ET for 30 min partially attenuated the inhibition of BiP and CHOP by adenoviral PLIN5 overexpression at either transcriptional or translation level and rescue the mRNA level of *XBP-1(s)*. Furthermore, overexpressing CPT-1A in PLIN5 deficient cells alleviated the induction of BiP, CHOP by PA incubation (Fig. 6c). The protective role of CPT-1A overexpression was also observed in decreasing cell apoptosis and increasing cell viability (Fig. 6d-e). Taken together, these data indicate that upregulation of PLIN5 may attenuate PA-induced ER stress partly by promoting FAO in INS-1 β-cells.

## Discussion

It is well accepted that prolonged exposure to fatty acid in β-cells decreases insulin secretion and causes β-cell apoptosis ex vivo [35, 36]. This “lipotoxicity” is deemed as one of the culprits for the development of type 2 diabetes [3]. However, the molecular mechanisms underlying lipotoxicity remain incompletely unknown. In this study, we have examined the role of PLIN5 in protection against PA-induced β-cell lipotoxicity by using INS-1 β-cell lines and β-cell-specific PLIN5 overexpression mice. The data showed that PA-induced PLIN5 expression in vitro which resulted in increased LD formation. Consistently, PLIN5 protein expression in islets of HFD mice and ob/ob obesity mice significantly increased compared with that of control mice. Although lipid content was elevated in β-cells, upregulation of PLIN5 ameliorated metabolic stress-induced impairment in GSIS in INS-1 cells, which was confirmed in mice with targeted expression of PLIN5 in β-cells. Besides, cell viability and apoptosis were also significantly improved companied by accelerated ER stress resolution which was partially contributed by increased FAO in Ad-PLIN5 cells compared to Ad-GFP cells. Overall, our data show that PLIN5 is required to maintain the appropriate balance between fatty acid storage and FFA catabolism during periods of lipid overload and, thus, contributes to blocking lipotoxicity in β-cells.

Enhanced FAO stimulated by upregulation of PLIN5, followed by decreased ER stress may a major mechanism responsible for decreased lipotoxicity observed in the present study. Excess FAO stimulated by cytosolic FFA is commonly viewed as an inducer of increased oxygen consumption and oxidative stress that can theoretically lead to lipotoxicity [17, 37]. However, it was also proposed that enhanced lipid metabolism including FAO attenuated lipotoxicity in pancreatic β-cells [34]. Moreover, it is reported that elevation of FAO is associated with the protection role of PPARδ on PA-induced ER stress, one of the important risk factors for lipotoxicity, in INS-1 cells [33]. Given the possible role of PLIN5 in modulating ER stress and FAO in liver and skeletal muscle [17, 19, 20], we assessed whether the anti-lipotoxicity role of PLIN5 in pancreatic β-cells was partially attributed to the alleviation of ER stress by inducing FAO. As expected, overexpression of PLIN5 inhibited overnutrition-induced ER stress in INS-1 cells indicated by blocking ER stress markers, which is similar to previous data from liver [17]. While, inconsistent with our results, there were also reports demonstrated that PLIN5 deficiency in whole body had no effect on ER stress and muscle-specific deletion of PLIN5 reduced ER stress in skeletal muscle under the condition of chronic overnutrition [19, 20]. This apparent discrepancy implied that PLIN5 may exert tissue-specific regulation in target tissues, a premise which was also presented by a recent study [20]. We further proved that increased FAO was indispensable for PLIN5-mediated ER stress alleviation by inhibiting FAO (ET, an inhibitor of FAO) and inducing FAO (overexpression of CPT-1A) in INS-1 β-cells. The protective role of enhanced FAO was also indicated as decreased cell apoptosis and elevated cell viability. The detailed mechanism of ER stress augmentation by inhibition of FAO was not clear and remains to be studied in the future. We speculated that accumulation of lipid intermediates resulting from inhibition of FAO could render β-cells susceptible to PA-induced toxicity. Indeed, previous studies have revealed the link between PLIN5, lipid metabolism and ER homeostasis [17, 20].

Lipid storage alone, opposite to lipid catabolism, may also contribute to decrease lipotoxicity in non-adipose tissues. It has been proposed that reduced intracellular level of PA stored in LD in the form of TG is less toxic than PA alone to pancreatic β-cells [38]. The ability of normal β-cells to form and accumulate cytoplasmic TGs may protect against PA-induced apoptosis by inhibiting a cellular rise in toxic free fatty acyl moieties [38]. Our data support this opinion. In the present study, PLIN5 overexpression increased the ability of β-cells to store LD detected by Nile red staining after 12 h PA treatment possibly by inhibiting lipolysis. Accordingly, these cells presented alleviated lipotoxicity manifested by improved GSIS, cell viability and decreased apoptosis compared with INS-1 cells treated with PA alone. Similarly, the regulations of lipid storage and lipotoxicity by PLIN5 were also observed in peripheral tissues such as skeletal muscle, heart and liver [14, 17, 18]. However, the causal link between PLIN5 overexpression-induced LD accumulation and anti-lipotoxicity role can possibly be verified by blocking LD storage via gain and loss of function of key enzymes involved in lipid metabolism and needs to be investigated in these tissues in the future.

It has been reported that the mRNA level of PLIN5 was not affected by overnight oleic acid or PA incubation in human islets and MIN6 cells [21], while the level of PLIN5 mRNA was upregulated significantly by acute PA treatment (1 h) and gradually decreased to the level of baseline in INS-1 cells, suggesting that the effect of FFA on the expression of PLIN5 is time-dependent. It is consistent with the data from Trevino et al. who found that PLIN5 mRNA expression was increased after 6 h of fasting and slowly declined after refeeding in mouse islets, as we know the circulating FFAs increase during fasting and decrease after feeding [21, 39]. Thus, PLIN5 may serve as a LD-derived metabolic demand sensor to play different roles in kinds of cellular molecular process in β-cells. Indeed, in a short-term PA treatment model (1 h), PLIN5 was verified to partition of FA into LD that is released upon refeeding, and hence aid postprandial insulin secretion in PKA-cAMP- and GPR40-dependent manners [21]. Besides, GLP-1R pathway which plays a major role in promoting insulin secretion postprandially and activates PKA was also confirmed to involve in the regulation of insulin secretion mediated by PLIN5 [21]. Consistently, we also observed the enhanced GSIS in Ad-PLIN5 β-cells compared with Ad-GFP control cells in the context of 12 h PA treatment. And the expression of GLP-1R was significant induced by overexpressing PLIN5 in INS-1 β-cells. Although impaired GSIS under chronic overnutrition (48 h PA treatment) was partially rescued due to the possible protective role of PLIN5 against lipotoxicity in our in vivo and in vitro study, the effect of PLIN5 on insulin secretion-associated pathways such as PKA-cAMP, GPR40, and GLP-1R may also contribute to the GSIS improvement. Recently, muscle-specific deletion of PLIN5 in mice has been shown to result in decreased circulating insulin and C-peptide levels because of reduced circulating FGF21, a myokine which is known to improve pancreatic insulin secretion [20]. Hence, improved GSIS in AAV-PLIN5 mice may also contributed by altered PLIN5 expression in muscle which is mild elevated in the present study. Whether β-cell-specific overexpression of PLIN5 interfere the circulating level of FGF21 and plays a role in pancreas-muscle crosstalk need to be investigated in the future.

We described here PLIN5 as a PPARs target gene in INS-1 cells, consistent with that in muscle and liver [25, 26, 40]. Identifying the regulatory factors of PLIN5 is helpful in understanding its biological roles. Activation of PPARβ/δ was reported to protect pancreatic β-cells from PA-induced apoptosis by upregulating the expression of GLP-1R [41]. And our data showed that PPARβ/δ induced robust upregulation of PLIN5 indirectly, while PLIN5 induced dramatic increase of GLP-1R, it is reasonable to speculate that the anti-lipotoxicity action of PPARβ/δ may involve its regulation of PLIN5 and GLP-1R. In favor of the role of PLINs in lipid metabolism, most PLIN genes including *PLIN5* are transcriptionally regulated by PPARs. Under HFD, we observed a significant upregulation of PLIN5 protein levels in pancreatic β-cells. It is still unclear how HFD regulates β-cell PLIN5 expression. FFAs, rich in HFD, are also natural ligands that increase PPARs transcriptional activity and may thus lead to upregulation of PLIN5. We speculated that upregulation of PLIN5 mediated by HFD seems to be an adaptive mechanism to favor FFA sequestering and accumulation into TG pools and thus relieve lipotoxicity.

## Conclusion

We have demonstrated for the first time that PLIN5 plays a critical role in the protection of pancreatic β-cells from lipotoxicity-induced cellular dysfunction. This protection appears to be partially mediated by alleviated ER stress, consequent to enhanced FAO. Besides, induction of lipid storage and GLP-1R expression by PLIN5 may also play a role in the process. These findings are significant in T2DM as lipotoxicity is often associated with the pathogenesis of this type of diabetes. A decrease in PLIN5 protein level in β-cells may contribute to the T2DM development and targeting PLIN5 may be useful for therapeutic development in this disease.

## Additional file

Additional file 1: **Figure S1.** The map and sequences of AAV-PLIN5 and AAV-MIP. The AAV2/8 vector contains mouse insulin promoter (MIP) and GFP, with sites for ECORI, BamHI, NdeI, SacI and ApaI. The size of AAV2/8 vector, MIP and PLIN5 was 4,492 bp, 874 bp and 1,392 bp respectively. Left: map of AAV-PLIN5; Right: map of AAV-MIP. Sequences of MIP: 5′-TTGTAGCTGGAATAGAGCATGCACTAACAGATGGAGACAGCTGGCTTTGAGCTCTGAAGCAAGTATTACATATGGAGACTTGCTGGCCTTCAGGTGCTTATCTTGTTATTGGATACTGCAGGAGGATGTACCACAGGGCTTCAGCTCAGCTGACCCCCAAGTGGGATATGGAAAGAGAGATAGAGGAGGAGGGACCATTAAGTGCCTTGCTGCCTGAATTCTGCTTTCCTTCTACCTCTGAGAGAGAGCTGGGGACTCGGCTGAGTTAAGAACCCAGCTATCAATTGGAACTGTGAAACAGTCCAAGGGACAAAGATACTAGGTCCCCAACTGCAACTTCCTGGGGAATGATGTGGAAAAATGCTCAGCCAAGGACAAAGAAAGCATCACCCACTCTGGAACAATGTCCCCTGCTGTGAACTGGTTCATCAGGCCATCAGGGCCCCTTGTTAAGACTCTAATTACCCTAGGACTAAGTAGAGGTGTTGACGTCCAATGAGCGCTTTCTGCAGACCTAGCACCAGGGAAGTGTTTGGAAACTGCAGCTTCAGCCCCTCTGGCCATCTGCTGACCTACCCCACCTGGAGCCCTTAATGGGTCAAACAGCAAAGTCCAGGGGGCAGAGAGGAGGTGCTTTGGTCTATAAAGGTAGTGGGGACCCAGTAACCACCAGCCCTAAGTGATCCGCTACAATCAAAAACCATCAGCAAGCAGGAAGGTACTCTTCTCAGTGGGCCTGGCTCCCCAGCTAAGACCTCAGGGACTTGAGGTAGGATATAGCCTCCTCTCTTACGTGAAACTTTTGCTATCCTCAACCCAGCCTATCTTCCAGGTTATTGTTTCAACA-3′ Sequences of PLIN5: 5′-ATGTCTGAAGAAGAGGCGGCTCAGATCCCCAGATCCAGTGTGTGGGAGCAGGACCAGCAGAACGTGGTGCAGCGTGTGGTGGCTCTGCCCCTGGTCAGGGCCACGTGCACCGCGGTCTGCGATGTTTACAGTGCAGCCAAGGACAGGCACCCGCTGCTGGGCTCCGCCTGCCGCCTGGCTGAGAACTGCGTGTGCGGCCTGACCACCCGTGCCCTGGACCACGCCCAGCCGCTGCTCGAGCACCTGCAGCCCCAGCTGGCCACTATGAACAGCCTCGCCTGCAGGGGCCTGGACAAGCTGGAAGAGAAGCTTCCCTTTCTCCAGCAACCTTCGGAGACGGTGGTGACCTCAGCCAAGGACGTGGTGGCCAGCAGTGTCACGGGTGTGGTGGACCTGGCCCGGAGGGGCCGGCGCTGGAGCGTGGAGCTGAAGCGCTCCGTGAGCCATGCTGTGGATGTTGTACTGGAAAAATCAGAGGAGCTGGTGGATCACTTCCTGCCCATGACGGAGGAAGAGCTCGCGGCACTGGCGGCTGAGGCTGAAGGCCCTGAAGTGGGTTCGGTGGAGGATCAGAGGAGACAGCAGGGCTACTTTGTGCGCCTCGGCTCCCTGTCAGCACGGATCCGCCACCTGGCCTACGAGCACTCTGTGGGGAAACTGAGGCAGAGCAAACACCGTGCCCAGGACACCCTGGCCCAGCTGCAGGAGACGCTGGAGCTGATAGACCACATGCAGTGTGGGGTGACCCCCACCGCCCCGGCCCGCCCTGGGAAGGTGCACGAGCTGTGGGGGGAATGGGGCCAGCGCCCTCCGGAGAGCCGCCGCCGGAGCCAGGCAGAGCTGGAGACGCTGGTGCTGTCCCGCAGCCTGACCCAGGAGCTGCAGGGCACGGTAGAGGCTCTGGAGTCCAGCGTGTGGGGCCTGCCCGCCGGCGCCCAGGAGAAGGTGGCTGAGGTGCGGCGCAGTGTGGATGCCCTGCAGACCGCCTTCGCTGATGCCCGCTGCTTCAGGGACGTGCCAGCGGCCGCGCTGGCCGAGGGCCGGGGTCGCGTGGCCCACGCGCACGCCTGCGTGGACGAGCTGCTGGAGCTGGTGGTGCAGGCCGTGCCGCTGCCCTGGCTGGTGGGACCCTTCGCGCCCATCCTTGTGGAGCGACCCGAGCCCCTGCCCGACCTGGCGGACCTGGTGGACGAGGTCATCGGGGGCCCTGACCCCCGCTGGGCGCACCTGGACTGGCCGGCCCAGCAGAGAGCCTGGGAGGCAGAGCACAGGGACGGGAGTGGGAATGGGGATGGGGACAGGATGGGTGTTGCCGGGGACATCTGCGAGCAGGAACCCGAGACCCCCAGCTGCCCGGTCAAGCACACCCTGATGCCCGAGCTGGACTTCTGA-3′ (EPS 980 kb)

## Data Availability

The datasets used and analyzed during the current study are available from the corresponding author on reasonable request.

## Abbreviations

AAV-MIP: Control adeno-associated virus
AAV-PLIN5: Adeno-associated virus overexpressing PLIN5
Ad-GFP: Control adenovirus carrying green fluorescent protein
Ad-PLIN5: Recombinant adenovirus encoding full-length rat PLIN5
ATF6: Transcription factor 6
AUC_g_: Areas under the curves for blood glucose in glucose tolerance tests
AUC_itt_: Areas under the curves for blood glucose in insulin tolerance tests
BiP: Immunoglobulin heavy chain-binding protein
CCK-8: Cell Counting Kit-8
CHOP: C/EBP homologous protein
CPT-1: Carnitine palmitoyltransferase 1
ER: Endoplasmic reticulum
ET: Etomoxir
FAO: Fatty acid oxidation
FBS: Fetal bovine serum
FFA: Free fatty acids
GFP: Green fluorescent protein
GLP-1R: Glucagon-like peptide-1 receptor
GSIS: Glucose-stimulated insulin secretion
GTT: Glucose tolerance tests
HFD: High fat diet
ITT: Insulin tolerance tests
KRB: Krebs-Ringer bicarbonate
LD: Lipid droplet
M-MLV: Moloney murine leukemia virus
MOI: Multiplicity of infection
PA: Palmitate
PBS: Phosphate-buffered saline
PERK: Protein kinase RNA-like endoplasmic reticulum kinase
PLIN5: Perilipin 5
PLINs: Perilipin family proteins
Q-PCR: Quantitative real-time RT-PCR
SREBP-1c: Sterol-regulatory element binding protein-1c
T2DM: Type 2 diabetes; TG: Triglyceride
UPR: Unfolded protein response
XBP-1(s): Spliced XBP-1
